# Repeated plantar electrical stimulation is associated with altered lower-limb somatosensory processing in older adults: evidence from somatosensory evoked potentials

**DOI:** 10.3389/fnagi.2026.1939284

**Published:** 2026-09-18

**Authors:** Aoi Mase, Risa Tashiro, Kanoko Ito, Ryoji Kasanami, Ryota Sakurai, Chiaki Ohtaka, Manabu Shibasaki, Hiroki Nakata

**Affiliations:** 1 Graduate School of Humanities and Sciences, Nara Women’s University, Nara, Japan; 2 Faculty of Engineering, Nara Women’s University, Nara, Japan; 3 Faculty of Education, Health and Sports Science Education, Nara University of Education, Nara, Japan; 4 Research Team for Social Participation and Healthy Aging, Tokyo Metropolitan Institute for Geriatrics and Gerontology, Tokyo, Japan

**Keywords:** aging, electric stimulation, electroencephalography, SEPs, somatosensory processing

## Abstract

**Introduction:**

Age-related declines in lower-limb somatosensory function may contribute to impaired motor control and increased fall risk in older adults. Although plantar sensory stimulation has been reported to improve postural control, its effects on neural processing remain unclear. This study investigated whether repeated plantar electrical stimulation modifies lower-limb somatosensory processing in older adults using somatosensory evoked potentials (SEPs).

**Methods:**

83 older adults aged ≥65 years were assigned to either a plantar electrical stimulation group (PES, *n* = 37) or a non-intervention control group (CON, *n* = 46). The PES group performed home-based plantar electrical stimulation for 8 weeks (30 min/session, ≥2 days/week). SEPs elicited by tibial, peroneal, and median nerve stimulation were recorded before and after the intervention. SEP latencies and amplitudes were analyzed using mixed-design ANOVA, and ANCOVA was performed for components showing baseline group differences.

**Results:**

In the PES group, P37 latency elicited by tibial nerve stimulation and P37 and N50 latencies elicited by peroneal nerve stimulation were shorter at Post than at Pre, whereas no significant changes were observed in the CON group. ANCOVA confirmed that the group effect remained significant for P37 latency during tibial and peroneal nerve stimulations after adjustment for baseline values. Significant negative correlations were observed between baseline latencies and intervention-related changes, although these findings should be interpreted cautiously because change scores include baseline values. No significant intervention effects were observed for SEP amplitudes during lower-limb stimulation, and median nerve SEPs showed no comparable latency shortening.

**Conclusion:**

Repeated plantar electrical stimulation was associated with shortened early SEP latencies elicited by lower-limb nerve stimulation. No comparable latency shortening was observed in median nerve SEPs, suggesting that the observed changes were more prominent in lower-limb than in upper-limb somatosensory processing. These findings suggest that repeated plantar electrical stimulation may be associated with changes in the temporal characteristics of lower-limb somatosensory processing in older adults. Further studies should determine whether these neurophysiological changes are associated with improvements in balance, mobility, and other functional outcomes.

## 1 Introduction

Falls in older adults constitute a critical health issue because they can lead to injuries, long-term care needs, and death. According to the Vital Statistics of Japan, numbers of fall-related deaths among older adults exceed traffic-related fatalities, with most incidents resulting from same-level falls caused by slipping, tripping, or loss of balance. These data suggest that impairments in foot placement and postural control may contribute to an increased risk of falls and related injuries in older adults (Ministry of Health, Labour and Welfare, 2018). Similarly, pedal misapplication among older drivers remains a significant societal issue. Brake-accelerator confusion occurs markedly more frequently in drivers aged ≥75 years than in younger drivers (National Police Agency, Traffic Bureau, 2024). Although falls and driving accidents occur in different contexts, both depend on the ability to appropriately perceive sensory information from the lower limbs and generate timely motor responses. Therefore, maintaining lower-limb somatosensory function may be important for preserving sensorimotor function in older adults.

Somatosensory sensitivity declines with aging and is influenced by the skin condition; declines are more pronounced in the lower than upper limbs, with reduced sensitivity also observed in deep sensation (Lin et al., 2005; Johnson et al., 2021), and vibration perception thresholds increase with age, particularly in the lower limbs (Perret and Regli, 1970). Jacobs and Love (1985) showed in the human sural nerve that degeneration and regeneration coexist at an older age in both myelinated and unmyelinated fibers, and that the somatosensory conduction velocity is reduced, suggesting compromised consistency and reliability of sensory input. Because sensory information from the lower limbs must travel a longer distance to reach the central nervous system than that from the upper limbs, age-related slowing of peripheral nerve conduction may have a greater impact on the timing of sensorimotor processing. Furthermore, lower-limb sensory input contributes to sensorimotor processing during standing and walking. Therefore, even small delays or inaccuracies in peripheral sensory information may affect motor control.

Recent intervention studies suggest that enhancing plantar sensory input may improve balance and mobility in older adults (Hsu et al., 2022; Oddsson et al., 2022; Cavlak et al., 2025). For example, Hsu et al. (2022) found that 6 months of mechanical tactile stimulation to the lower leg using a sensory substitution device (Walkasins) in older adults with peripheral neuropathy improved gait function and altered functional connectivity in sensorimotor, frontoparietal, and default mode networks. Cavlak et al. (2025) reported that an eight-week plantar sensory exercise intervention in older adults aged ≥70 years improved static balance and reduced fall risk. Collectively, these findings suggest that plantar somatosensory input contributes to lower-limb sensorimotor function, and that maintaining or enhancing plantar sensory function may support functional mobility in older adults. Beyond improvements in functional performance, repetitive sensory electrical stimulation has been shown to induce use-dependent plasticity within the somatosensory system, resulting in improved tactile discrimination and altered cortical reorganization in the upper limb (Pleger et al., 2003). Whether similar neuroplastic adaptations can be induced by repeated plantar electrical stimulation remains unknown.

Neural mechanisms of lower-limb somatosensory processing have been examined using time-locked averaging electroencephalography (EEG) and magnetoencephalography (MEG). Using posterior tibial nerve stimulation, Hari et al. (1996) reported temporal and spatial characteristics of somatosensory evoked magnetic fields (SEFs) that were distinct from those elicited by median nerve stimulation. They suggested that early components mainly involve Brodmann areas 1, 3a, and 5 rather than area 3b. Valeriani et al. (2001) utilized somatosensory evoked potentials (SEPs) with posterior tibial nerve stimulation in patients implanted with deep electrodes for drug-resistant Parkinson’s disease. They suggested that lower-limb SEPs, including the earliest P37, reflected neural activity across a broader sensorimotor integration network (including motor-related regions) rather than localized activity restricted to the primary somatosensory cortex, and that later components from N50 onward involved cortico–cortical interactions and higher-order processing. Kakigi (1987) reported marked latency prolongation of short-latency components in older adults, indicating age-related slowing of conduction at peripheral, spinal, and subcortical levels, whereas long-latency components tended to show larger amplitudes, suggested to reflect relatively increased cortical activity or reduced inhibitory mechanisms; these changes were particularly marked after age 75.

Although previous intervention studies demonstrated beneficial effects of plantar sensory stimulation on balance, gait performance, and brain functional connectivity, it remains unclear whether repeated plantar electrical stimulation modifies neural transmission within the lower-limb somatosensory pathway. Consequently, the neurophysiological mechanisms underlying these functional improvements remain poorly understood. Previous studies characterized the cortical generators of lower-limb SEPs and demonstrated age-related changes in SEP responses; however, evidence regarding intervention-related modifications of lower-limb SEP responses in older adults remains limited. SEPs provide a direct and non-invasive measure of neural transmission and cortical processing within the somatosensory system, and may therefore be useful for evaluating intervention-related changes in somatosensory processing.

Based on this background, the purpose of the present study was to evaluate the effects of an eight-week plantar electrical stimulation intervention on somatosensory function in older adults using SEPs. Because the intervention targeted the plantar surface of the foot, we expected any associated neurophysiological changes to be more apparent in pathways related to lower-limb somatosensory processing than in upper-limb pathways. We hypothesized that repeated plantar stimulation would be associated with shorter latencies of SEP components elicited by lower-limb afferent input. Furthermore, we examined whether such changes were more prominent in lower-limb SEPs elicited by posterior tibial and peroneal nerve stimulation than in median nerve SEPs.

## 2 Methods

### 2.1 Participants

83 older adults aged ≥65 years participated in this study and were allocated to either an intervention group receiving plantar electrical stimulation (PES group; *n* = 37; 10 males, 27 females) or a no-intervention control group (CON group; *n* = 46; 18 males, 28 females) based on their willingness to participate in the eight-week intervention. During recruitment, participants were informed about the study procedures and the duration of the intervention. Those who agreed to undertake the eight-week plantar electrical stimulation intervention were allocated to the PES group, whereas those who preferred not to undertake the intervention because of its duration or burden were allocated to the CON group. This participant preference-based allocation contributed to the unequal group sizes. SEP analyses were conducted separately for tibial, peroneal, and median nerve stimulation. Due to artifacts and poor waveform quality, some recordings were excluded from the analyses. Participants were right-handed and were verbally screened for conditions that could affect somatosensory function or SEP measurements. Exclusion criteria included a history of seizure disorders, head injury or neurosurgery, use of a cardiac pacemaker, concurrent participation in another intervention study, peripheral neuropathy, neurological disorders, and other sensory impairments. At the time of the experiment, participants were also verbally confirmed to have no knee or foot pain or knee- or foot-related conditions requiring ongoing medical care, and to have no physical conditions that would interfere with the experimental procedures. Prior to the experiment, cognitive status was assessed using the Mini-Mental State Examination (MMSE). Mean MMSE scores were 29.4 ± 0.8 in the PES group and 29.2 ± 1.1 in the CON group, suggesting that no marked cognitive impairment was present in either group. Habitual physical activity was assessed using the Global Physical Activity Questionnaire (GPAQ). Total physical activity was calculated as metabolic equivalent (MET)-minutes per week according to the GPAQ analysis guidelines. Participants received a detailed explanation of the study purpose and procedures in advance, and written informed consent was obtained prior to participation. The study protocol was approved by the Research Ethics Committee of Nara Women’s University (approval no. 24-40).

### 2.2 Study design and procedure

The experiment was conducted from January to April 2025 and from November 2025 to March 2026. The study included an eight-week intervention period. Somatosensory function was assessed before (Pre) and after (Post) the intervention by recording SEPs in both the PES and CON groups.

Experiments were performed in a temperature-controlled laboratory at 25 °C. In order to record SEPs, constant-current square-wave electrical pulses were delivered to the right tibial, peroneal, and median nerves using a stimulator integrated in the Neuropack X1 system (Nihon Kohden, Tokyo, Japan), which allows simultaneous electrical stimulation and EEG recording. For tibial nerve stimulation, bipolar surface electrodes were placed posterior to the medial malleolus. For peroneal nerve stimulation, electrodes were positioned over the common peroneal nerve at the fibular head. For median nerve stimulation, electrodes were positioned over the median nerve at the wrist (Figure 1). The stimulus duration was 0.2 ms, and approximately 250–300 stimuli were delivered at a frequency of 3 Hz while participants remained relaxed and were instructed not to consciously react to the stimuli (Mase et al., 2025). Stimulus intensity was individually adjusted to the motor threshold, defined as the minimum intensity required to elicit a visible muscle twitch. Specifically, stimulation intensity was set to produce a slight but clearly visible movement of the great toe during tibial nerve stimulation, ankle dorsiflexion during peroneal nerve stimulation, and thumb movement during median nerve stimulation. Actual stimulus intensities are presented in Table 1.

**Figure 1:**
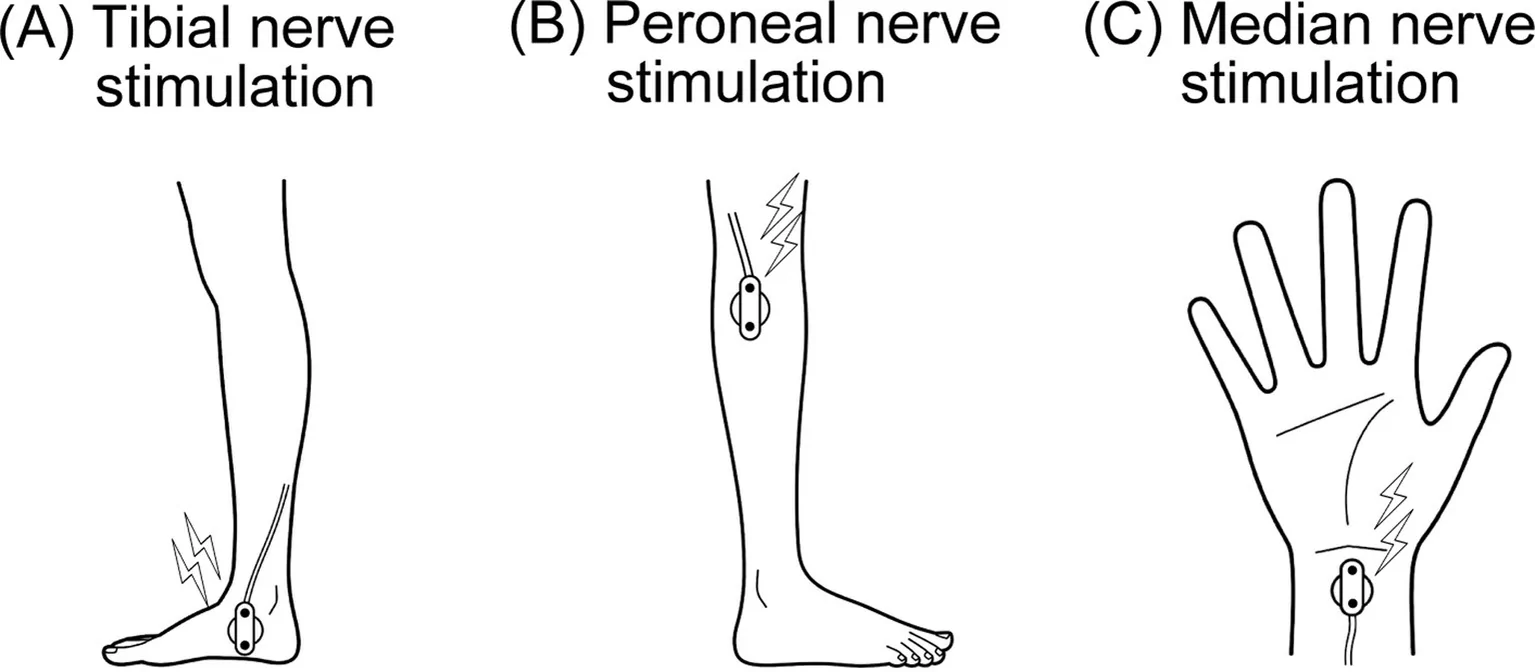
Electrical stimulation sites for somatosensory evoked potential (SEP) recordings. **(A)** Tibial nerve stimulation, **(B)** peroneal nerve stimulation, and **(C)** median nerve stimulation.

**Table 1:** Mean stimulus intensity (mA) at pre- and post-intervention in PES and CON with SD.

(mA)	PES		CON	
	Pre	Post	Pre	Post
Tibial nerve stimulation	10.2 (2.6)	10.2 (2.4)	10.8 (3.0)	10.4 (3.1)
Peroneal nerve stimulation	6.7 (3.4)	4.8 (1.9)^***^	6.6 (3.0)	5.9 (2.0)^***^
Median nerve stimulation	4.2 (1.5)	3.1 (1.2)	3.7 (1.1)	4.0 (0.8)

^***^p < 0.001 (vs. Pre). PES, plantar electrical stimulation group; CON, control group.

To ensure consistency between the Pre and Post assessments, all SEP recordings were conducted using the same experimental protocol and recording parameters. The locations of stimulation electrodes were documented photographically during the Pre assessment and reproduced as closely as possible during the Post assessment. Stimulus intensity was determined using the same motor-threshold criterion at both time-points. These procedures were implemented to minimize variability in electrode placement and stimulation conditions across sessions.

### 2.3 EEG recording and data analysis

EEG was recorded with Ag/AgCl disk electrodes placed on the scalp at Fz, Cz, Pz, and C3’ (C3’ was 2 cm posterior to C3), according to the International 10–20 System. Each scalp electrode was referenced to linked earlobes, which were mathematically calculated as an averaged reference. An electrooculogram (EOG) was recorded bipolarly using a pair of electrodes placed 2 cm lateral to the lateral canthus of the right eye and 2 cm above the upper edge of the right orbit to monitor eye movements and blinks. Epochs containing EOG activity exceeding ±100 μV were excluded from averaging. In addition, all raw EEG data were visually inspected offline, and epochs containing clear artifacts were excluded from averaging. Impedance was maintained at less than 5 kohm. All EEG signals were collected using a signal processor (Neuropack X1 system, Nihon-Kohden, Tokyo, Japan). For the recording of SEPs, the bandpass filter was 1–1,500 Hz. A 60-Hz notch filter was also applied to eliminate power-line interference. EEG epochs were recorded and analyzed from 10 ms before to 90 ms after stimulus onset (−10 to 90 ms), with stimulus onset defined as 0 ms. The sampling rate was 5,000 Hz. The peak amplitudes and latencies of individual SEP components were obtained using the measuring scale of the Neuropack system with visual inspection. Peak identification was performed without knowledge of group allocation or assessment time. All clearly identifiable SEP components were measured, whereas waveforms in which the corresponding SEP peaks could not be clearly identified were excluded from the analysis. Consequently, the number of participants included in the final analysis differed across nerve stimulation conditions.

For tibial and peroneal nerve stimulation, analyses focused on the Cz electrode, and additional electrodes were placed based on previous studies (Valeriani et al., 2001; Wasaka et al., 2005, 2006). Peak latencies and amplitudes at Cz were measured for P37, N50, P60, and N70 components. Amplitudes were measured using a peak-to-peak method for all components except P37. Because P37 was the first identifiable component, its amplitude was measured with a baseline-to-peak method.

For median nerve stimulation, we focused on Fz and C3’ electrodes, and the other electrodes were additionally placed based on previous studies (Mase et al., 2025; Nakata et al., 2021). We measured P12, N15, P18, and N30 components at Fz, and P12, N18, P22, N27, P45, and N60 components at C3’. Peak amplitudes were measured using a peak-to-peak method for all components except P12 at both electrodes, because P12 is the earliest identifiable component and reflects subcortical neural activity (Mase et al., 2025; Nakata et al., 2021). Therefore, the P12 amplitude was measured from the baseline to peak.

### 2.4 Eight-week intervention

During the eight-week period, participants in the PES group performed home-based plantar electrical stimulation using a low-frequency electrical stimulator (OMRON HV-F021/HV-F022-W certified medical device in Japan). Following the manufacturer’s instructions, one self-adhesive electrode was placed on the plantar surface of the foot, primarily covering the arch region, and the other was placed on the calf (Figure 2). Stimulation was applied bilaterally for 15 min per foot (total: 30 min/day). Participants were allowed to perform the stimulation at any time of day according to their daily schedule. The device output level could be adjusted across 15 steps, and participants were instructed to select an intensity they perceived as comfortable. A square-wave pulsed current with frequencies varying between 1 and 19 Hz was applied, with a maximum pulse width of 150 μs. The intervention was performed at least twice per week for 8 weeks. Participants recorded the date, duration, and stimulation intensity of each session on a monitoring sheet, which was collected after completion of the intervention. Because the interval between the pre- and post-intervention assessments varied slightly among participants due to scheduling constraints, the mean weekly stimulation frequency was calculated for each participant by dividing the total number of completed stimulation sessions by the actual intervention period in days and multiplying by seven. The CON group received no intervention and was instructed to maintain their usual daily activities throughout the same eight-week period.

**Figure 2:**
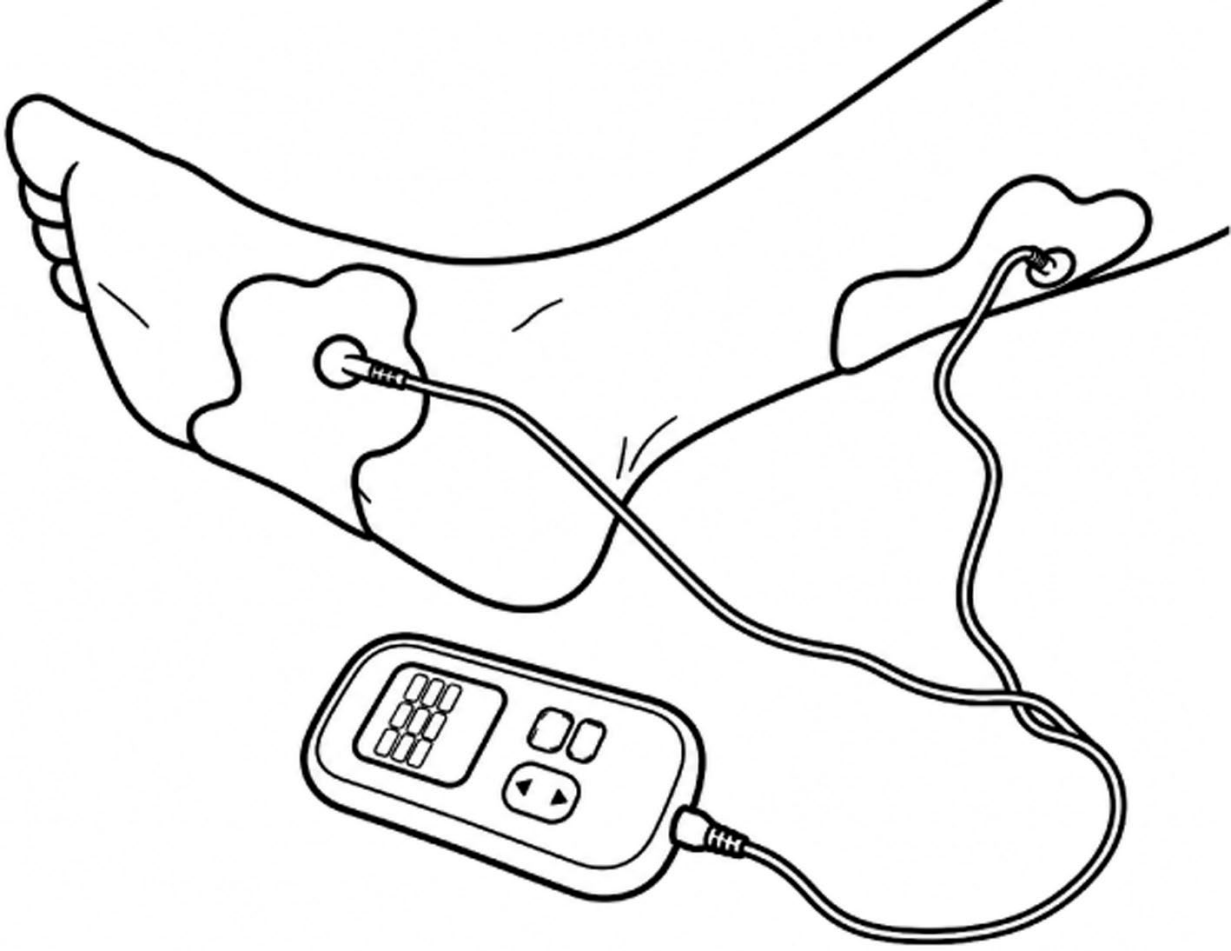
Electrode placement for plantar electrical stimulation during the eight-week intervention.

### 2.5 Statistical analysis

An *a priori* sample-size calculation was not performed. The initial sample size of 83 participants was determined based on the number of eligible participants who could be recruited during the study period and complete the experimental protocol. The final sample size differed among the nerve stimulation conditions because some SEP recordings were excluded due to artifacts or insufficient waveform quality.

Participant demographic characteristics (age, height, weight, BMI, MMSE, and GPAQ) were compared between the PES and CON groups for each SEP analysis. Continuous variables were compared using independent-samples t-tests when the assumptions for parametric testing were met; otherwise, the Mann–Whitney U test was used. When a significant between-group difference was identified in a demographic characteristic among participants included in the lower-limb SEP analyses, an additional repeated-measures analysis of covariance (ANCOVA) was performed for the corresponding lower-limb SEP outcome, with the demographic variable entered as a covariate, Group as the between-subject factor, and Time as the within-subject factor.

To examine the effects of the intervention, a two-way mixed-design analysis of variance (ANOVA) was separately conducted for each SEP outcome and for stimulation intensity during SEP recording, with Group (PES vs. CON) as the between-subject factor and Time (Pre vs. Post) as the within-subject factor. The primary effect of interest was the Group × Time interaction, indicating differential pre-to-post changes between groups. To account for multiple testing across lower-limb SEP outcomes, the Benjamini–Hochberg false discovery rate (FDR) correction was applied to the *p*-values of the Group × Time interactions for all SEP components elicited by tibial and peroneal nerve stimulation (*q* = 0.10), consistent with previous electrophysiological studies using this threshold (Killebrew et al., 2018; Cao et al., 2026). Median-nerve SEP outcomes were not included in this FDR family because they were assessed separately as upper-limb outcomes to examine whether changes extended beyond the stimulated lower-limb somatosensory system. For the lower-limb SEP outcomes subjected to FDR correction, Group × Time interactions with q < 0.10 were considered statistically significant. When a significant interaction was detected, post-hoc simple-effects analyses were performed to compare Pre vs. Post within each group and PES vs. CON at each time point. Assumptions for the mixed-design ANOVA were evaluated prior to hypothesis testing.

Because significant between-group differences were observed at baseline for some SEP outcomes, additional ANCOVAs were performed to account for these baseline differences when evaluating post-intervention group differences. In these analyses, the post-intervention value was entered as the dependent variable, Group (PES vs. CON) as the fixed factor, and the corresponding pre-intervention value as the covariate.

To examine whether stimulation intensity during SEP recording was associated with SEP measures, additional correlation analyses were conducted between baseline stimulation intensity and baseline SEP values for components showing significant intervention-related effects. In addition, correlations between changes in stimulation intensity during SEP recording (Post-Pre) and changes in the corresponding SEP measures (Post-Pre) were examined separately for the PES and CON groups.

Exploratory correlation analyses were also conducted between baseline SEP values (Pre) and their corresponding change scores (Post-Pre) to examine the association between initial values and subsequent changes. In addition, to examine whether intervention dosage was associated with changes in SEP measures, additional correlation analyses were performed in the PES group for components showing intervention-related changes. The total number of intervention sessions and average stimulation intensity during the eight-week intervention were correlated with the corresponding changes in SEP measures (Post-Pre).

Prior to the correlation analyses, normality was assessed using the Kolmogorov–Smirnov test. Pearson’s product–moment correlation coefficients were calculated for variables that met the normality assumption, whereas Spearman’s rank correlation coefficients were used when normality was not confirmed. Homogeneity of variance for group comparisons was assessed using Levene’s test. Because multiple bivariate correlations were examined, the Benjamini–Hochberg FDR correction was applied to exploratorily control the Type I error rate (*q* = 0.10). Statistical analyses were conducted using SPSS (version 31.0 for Windows; IBM Corp.), and the significance level was set at *p* < 0.05.

## 3 Results

Following artifact rejection, 27 participants (21 females and 6 males) from each group were included in the tibial nerve analysis, 28 participants (22 females and 6 males) from each group in the peroneal nerve analysis, and 31 participants (25 females and 6 males) at Fz and 33 (26 females and 7 males) participants from each group at C3’ in the median nerve analysis.

### 3.1 Participant characteristics

Participant characteristics for each SEP analysis are presented in Table 2. Significant between-group differences were observed in height among participants included in the tibial nerve analysis (*p* < 0.05) and in body weight among those included in the median nerve analysis at Fz (*p* < 0.05), with higher values in the PES group. However, the magnitude of these between-group differences was relatively small. No significant between-group differences were observed for the other demographic characteristics. The mean number of intervention sessions and average stimulation intensity for each nerve stimulation condition are presented in Table 2.

**Table 2:** Demographic characteristics of participants in the PES and CON groups.

	PES	CON
Tibial nerve stimulation analysis (*n* = 27/group)		
Age (years)	70.6 (4.2)	71.8 (4.1)
Sex (female/male)	21/6	21/6
Height (cm)	159.7 (6.6)^*^	157.2 (8.6)
Weight (kg)	60.8 (8.8)	56.6 (10.8)
BMI (kg/m^2^)	23.8 (2.9)	22.8 (2.8)
MMSE (score)	29.3 (1.0)	29.3 (1.1)
GPAQ (MET-min/week)	4746.7 (7647.1)	3781.5 (3079.0)
Total number of intervention sessions	46.1 (19.1)	–
Average intervention intensity (level)	11.4 (2.0)	–
Peroneal nerve stimulation analysis (*n* = 28/group)		
Age (years)	70.8 (4.1)	71.8 (4.1)
Sex (female/male)	22/6	22/6
Height (cm)	159.5 (6.3)	156.9 (8.4)
Weight (kg)	59.7 (8.9)	56.6 (9.3)
BMI (kg/m^2^)	23.5 (3.1)	22.9 (2.6)
MMSE (score)	29.4 (1.0)	29.1 (1.2)
GPAQ (MET-min/week)	4937.1 (7513.9)	3845.0 (3577.2)
Total number of intervention sessions	45.8 (17.1)	–
Average intervention intensity (level)	11.3 (2.0)	–
Median nerve stimulation analysis (Fz) (*n* = 31/group)		
Age (years)	70.8 (4.1)	72.2 (3.8)
Sex (female/male)	25/6	25/6
Height (cm)	159.8 (6.9)	156.4 (6.7)
Weight (kg)	59.8 (8.6)^*^	55.2 (9.0)
BMI (kg/m^2^)	23.5 (3.5)	22.5 (2.9)
MMSE (score)	29.4 (0.9)	29.1 (1.2)
GPAQ (MET-min/week)	4292.3 (7169.6)	4415.2 (3993.9)
Total number of intervention sessions	47.5 (17.9)	–
Average intervention intensity (level)	11.4 (1.9)	–
Median nerve stimulation analysis (C3’) (*n* = 33/group)		
Age (years)	70.9 (4.1)	72.6 (4.1)
Sex (female/male)	26/7	26/7
Height (cm)	159.6 (6.7)	156.6 (7.6)
Weight (kg)	59.5 (8.5)	55.2 (9.1)
BMI (kg/m^2^)	23.4 (3.4)	22.4 (2.8)
MMSE (score)	29.4 (0.9)	29.1 (1.2)
GPAQ (MET-min/week)	4432.1 (6983.1)	4010.0 (3307.3)
Total number of intervention sessions	47.6 (17.9)	–
Average intervention intensity (level)	11.4 (2.0)	–

^*^p < 0.05. PES, plantar electrical stimulation group; CON, control group.

### 3.2 Intervention adherence

Participants in the PES group completed a mean of 46.0 ± 18.1 stimulation sessions during the intervention period (range, 21–90 sessions). 27 of 37 participants (73.0%) met the minimum protocol criterion of completing at least two stimulation sessions per week during each of the eight intervention weeks. The mean stimulation frequency was 4.82 ± 1.95 sessions/week.

### 3.3 SEP in pre-post changes following tibial nerve stimulation

Figure 3 presents grand-averaged SEP waveforms for tibial, peroneal, and median nerve stimulation across all participants for each group, respectively. Tables 3–5 show the mean values of latencies and amplitudes of SEP for tibial, peroneal, and median nerve stimulation, respectively.

**Figure 3:**
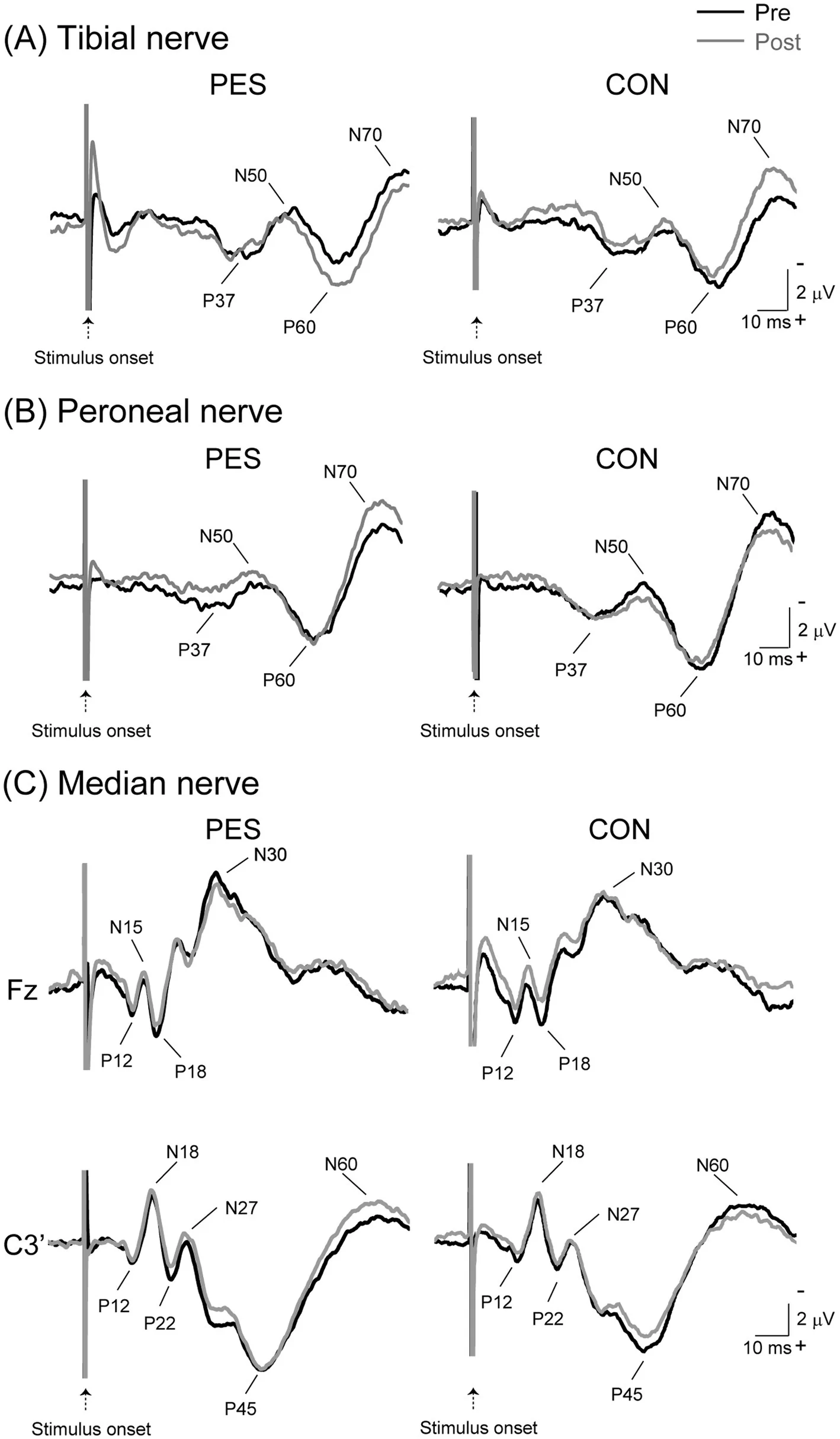
Grand-averaged SEP waveforms for **(A)** tibial nerve stimulation; **(B)** peroneal nerve stimulation; **(C)** median nerve stimulation. PES, plantar electrical stimulation group; CON, control group. The marked difference in the averaged SEP waveforms immediately after stimulus onset in the PES group may reflect differences in stimulation intensity between the pre and post measurements.

**Table 3:** The mean (SD) of latencies and amplitudes of SEP for tibial nerve stimulation.

	PES		CON	
	Pre	Post	Pre	Post
Latency (ms)				
P37	44.3 (3.7)^###^	41.6 (4.7)^***^	40.5 (3.5)	41.7 (3.5)
N50	57.6 (4.1)	55.0 (6.0)	54.4 (4.1)	54.4 (4.7)
P60	70.5 (4.6)	70.7 (5.5)	68.6 (5.0)	67.9 (5.6)
N70	85.0 (3.3)	85.2 (2.7)	83.7 (3.9)	83.0 (3.4)
Amplitude (μV)				
P37	1.6 (1.0)	1.3 (1.2)	1.2 (1.1)	1.3 (1.2)
N50	2.4 (1.3)	2.1 (1.2)	1.8 (1.2)	2.0 (1.0)
P60	2.8 (1.6)	2.7 (1.2)	2.8 (1.2)	2.8 (1.0)
N70	3.6 (2.8)	3.1 (1.4)	3.5 (2.0)	3.9 (2.0)

^***^p < 0.001 (vs. Pre), ^###^*p* < 0.001 (vs CON). PES, plantar electrical stimulation group; CON, control group.

**Table 4:** The mean (SD) of latencies and amplitudes of SEP for peroneal nerve stimulation.

	PES		CON	
	Pre	Post	Pre	Post
Latency (ms)				
P37	40.9 (3.5)^##^	37.6 (3.5)^***^	37.5 (4.1)	37.8 (3.8)
N50	51.9 (5.6)^##^	47.6 (5.6)^***^	48.3 (3.7)	49.5 (4.6)
P60	65.6 (4.3)	64.3 (5.1)	63.4 (3.8)	63.6 (4.4)
N70	83.1 (4.5)	83.1 (5.3)	81.5 (4.9)	81.5 (4.3)
Amplitude (μV)				
P37	1.1 (0.9)	1.2 (1.1)	1.1 (1.0)	1.3 (1.1)
N50	1.8 (1.6)	2.1 (1.4)	1.7 (1.1)	1.5 (0.9)
P60	2.8 (1.6)	3.1 (2.0)	3.0 (2.1)	2.8 (1.8)
N70	4.1 (2.7)	4.6 (3.1)	5.0 (4.1)	4.8 (4.1)

^***^p < 0.001 (vs. Pre) ^##^*p* < 0.01, ^###^*p* < 0.001 (vs. CON). PES, plantar electrical stimulation group; CON, control group.

**Table 5:** The mean (SD) of latencies and amplitudes of SEP for median nerve stimulation.

	PES		CON	
	Pre	Post	Pre	Post
Latency (ms)				
Fz				
P12	13.8 (1.1)	13.5 (0.9)	13.4 (1.1)	13.3 (1.0)
N15	16.9 (1.2)	16.7 (1.0)	16.4 (1.2)	16.8 (1.1)
P18	20.7 (1.3)	20.6 (1.3)	20.4 (1.6)	20.2 (1.7)
N30	38.4 (4.0)	37.6 (3.3)	37.8 (4.3)	37.5 (3.0)
C3’				
P12	14.0 (1.3)	13.7 (1.1)	13.8 (1.4)	13.5 (1.1)
N18	19.6 (1.1)	19.6 (1.1)	19.3 (1.2)	19.3 (1.2)
P22	24.2 (1.4)	24.0 (1.3)	23.6 (1.6)	24.1 (1.6)
N27	28.3 (1.8)	28.2 (1.5)	28.6 (2.3)	28.1 (1.9)
P45	48.1 (5.8)	49.4 (5.1)	46.5 (6.8)	47.5 (6.3)
N60	68.3 (8.6)	72.6 (8.6)^*^	70.8 (7.9)	69.6 (7.7)
Amplitude (μV)				
Fz				
N15	1.5 (0.6)	1.6 (0.5)	1.6 (0.6)	1.6 (0.4)
P18	2.1 (0.9)	2.0 (0.9)	1.8 (0.8)	1.6 (0.7)
N30	4.5 (1.9)	4.2 (1.8)	4.1 (1.5)	3.6 (1.4)
C3’				
N18	3.1 (1.2)	3.1 (0.9)	2.8 (1.0)	2.8 (1.1)
P22	3.8 (1.9)	3.4 (1.6)	3.3 (1.4)	3.3 (1.6)
N27	2.1 (1.3)	1.8 (0.9)^**^	1.8 (1.1)	1.6 (0.9)^**^
P45	5.8 (3.2)	5.7 (2.8)	5.4 (2.3)	4.9 (1.7)
N60	7.0 (5.2)	7.3 (4.9)	7.4 (3.9)	6.4 (3.6)

^*^p < 0.05, ^**^p < 0.01 (vs. Pre). PES, plantar electrical stimulation group; CON, control group.

A significant Time × Group interaction was observed for both P37 latency (*F* (1, 52) = 7.353, *p* = 0.009, *η*^2^ = 0.105) and N50 latency (*F* (1, 52) = 4.838, *p* = 0.032, *η*^2^ = 0.085). Post-hoc analyses revealed that both P37 and N50 latencies were significantly shorter at Post than Pre in the PES group (both *p* < 0.001). In addition, the PES group exhibited significantly longer P37 and N50 latencies than the CON group at Pre (*p* < 0.001 and *p* = 0.006, respectively). After correction for multiple testing using the Benjamini-Hochberg FDR procedure, the interaction remained significant for P37 latency (*q* = 0.003), whereas the interaction for N50 latency did not survive the correction (*q* = 0.128).

To account for baseline differences, ANCOVA was additionally performed using baseline (Pre) latency as a covariate. For P37 latency, a significant main effect of baseline latency was observed (*F* (1, 51) = 18.085, *p* < 0.001, *η*^2^ = 0.262) and the main effect of Group remained significant after adjustment (*F* (1, 51) = 4.522, *p* = 0.038, *η*^2^ = 0.081). In contrast, although baseline latency significantly influenced N50 latency (*F* (1, 51) = 30.672, *p* < 0.001, *η*^2^ = 0.376), the main effect of Group was no longer significant after adjustment for baseline latency (*F* (1, 51) = 2.397, *p* = 0.128, *η*^2^ = 0.045; Figure 4).

**Figure 4:**
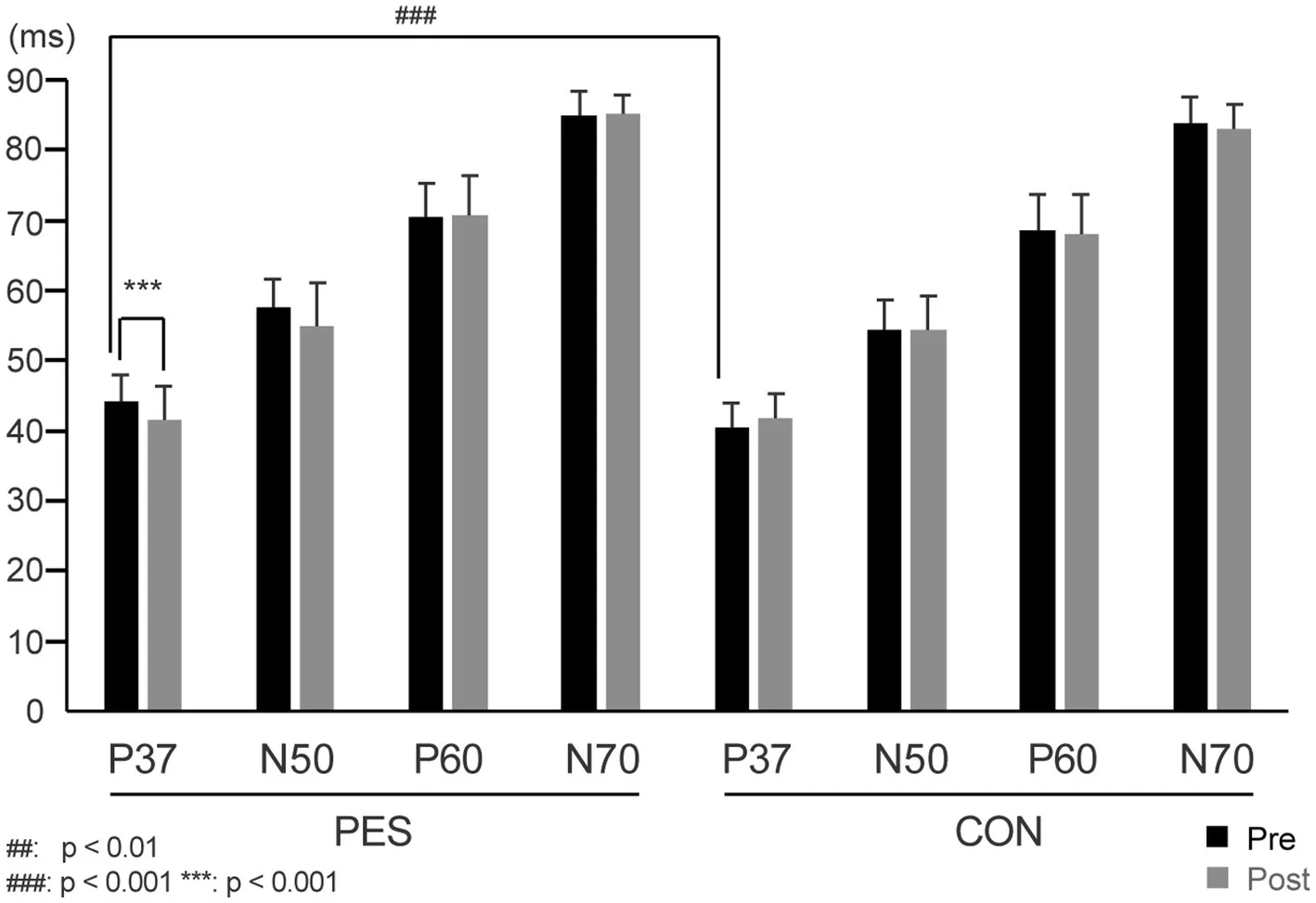
Mean latencies of SEP components during tibial nerve stimulation. PES, plantar electrical stimulation group; CON, control group. Asterisks indicate significant Pre-Post differences within the PES group for components showing a significant Time × Group interaction after FDR correction.

In addition, to account for height differences, ANCOVA was additionally performed using participants’ height as a covariate. The Time × Group interaction for P37 latency remained significant after adjustment for height (*F* (1, 51) = 14.586, *p* < 0.001, *η*^2^ = 0.222), whereas the Time × Height interaction was not significant (*F* (1, 51) = 0.796, *p* = 0.376, *η*^2^ = 0.015).

### 3.4 SEP in pre-post changes following peroneal nerve stimulation

A significant Time × Group interaction was observed for both P37 latency (*F* (1, 54) = 15.698, *p* < 0.001, *η*^2^ = 0.225) and N50 latency (*F* (1, 54) = 20.164, *p* < 0.001, *η*^2^ = 0.272). Post-hoc analyses revealed that both P37 and N50 latencies were significantly shorter at Post than Pre in the PES group (both *p* < 0.001). Furthermore, the PES group exhibited significantly longer P37 and N50 latencies than the CON group at Pre (*p* = 0.002 and *p* = 0.007, respectively). Both interactions remained significant after Benjamini-Hochberg FDR correction (q = 0.002 and 0.001, respectively) (Figure 5).

**Figure 5:**
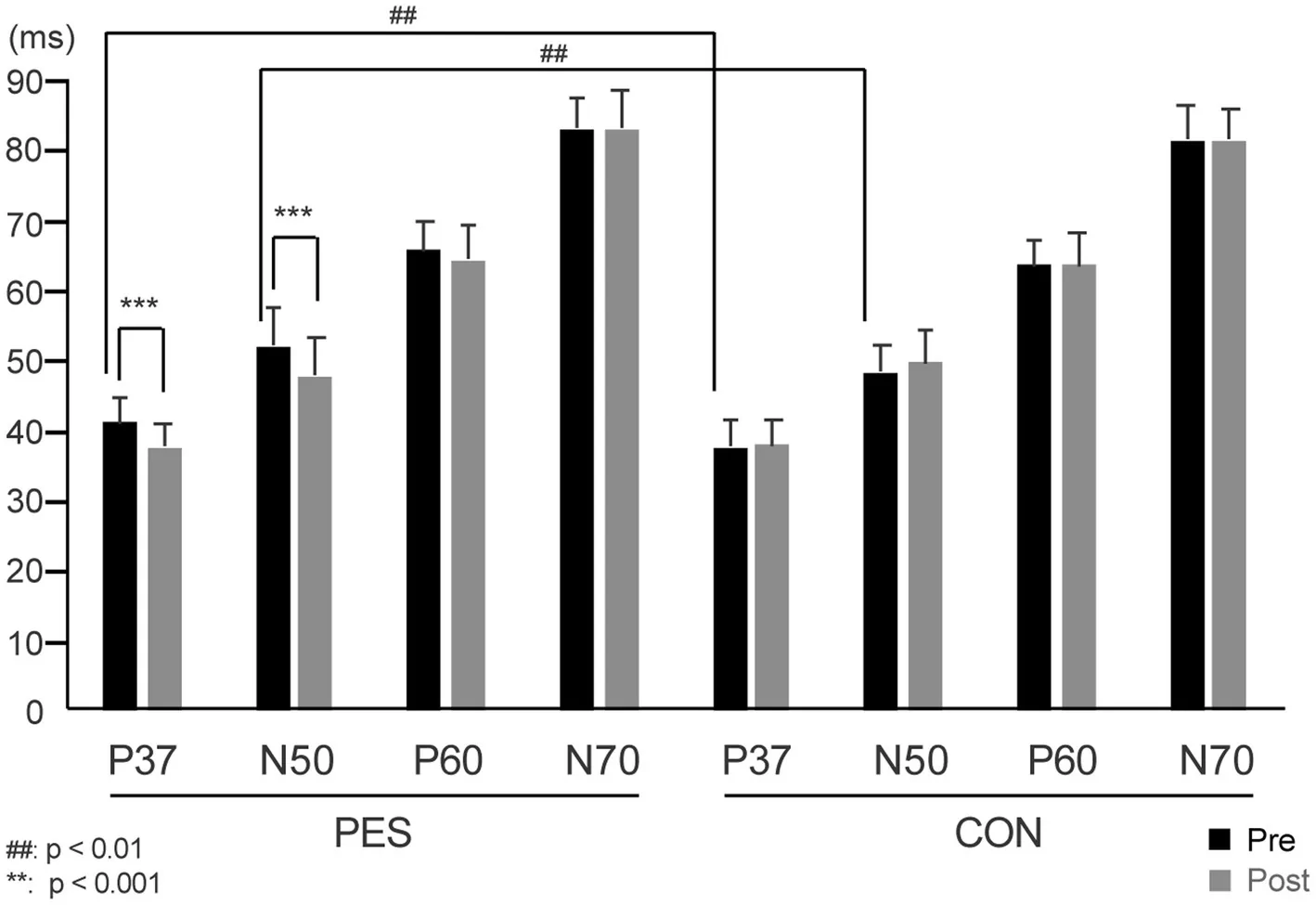
Mean latencies of SEP components during peroneal nerve stimulation. PES, plantar electrical stimulation group; CON, control group. Asterisks indicate significant Pre-Post differences within the PES group for components showing a significant *Time* × *Group* interaction after FDR correction.

ANCOVA for P37 latency showed significant main effects of baseline latency (*F* (1, 53) = 28.694, *p* < 0.001, *η*^2^ = 0.351) and Group (*F* (1, 53) = 5.830, *p* = 0.019, *η*^2^ = 0.099). Similarly, for N50 latency, significant main effects of baseline latency (*F* (1, 53) = 25.576, *p* < 0.001, *η*^2^ = 0.325) and Group (*F* (1, 53) = 11.566, *p* = 0.001, *η*^2^ = 0.179) were observed (Figure 5).

### 3.5 SEP in pre-post changes following median nerve stimulation

For N60 latency at C3’, a significant Time × Group interaction was recorded (*F* (1, 51) = 6.711, *p* = 0.012, *η*^2^ = 0.116). Post-hoc analyses revealed that N60 latency was significantly longer in Post in the PES group (*p* = 0.043). For N27 amplitude at C3’, a significant main effect of Time was observed (*F* (1, 63) = 7.353, *p* = 0.009, *η*^2^ = 0.105), with larger amplitudes noted at Pre than Post.

### 3.6 Association between baseline SEP latencies and intervention-related changes in PES

Correlation analyses were conducted for SEP components between baseline latencies (Pre) and intervention-related changes in latency (Post-Pre), including the P37 and N50 components elicited by tibial and peroneal nerve stimulation. A significant negative correlation was observed for P37 during tibial nerve stimulation (*r* = −0.385, *p* = 0.043). In addition, significant negative correlations were identified for the P37 and N50 components during peroneal nerve stimulation (*r* = −0.486, *p* = 0.014; *r* = −0.504, *p* = 0.007; Figure 6).

**Figure 6:**
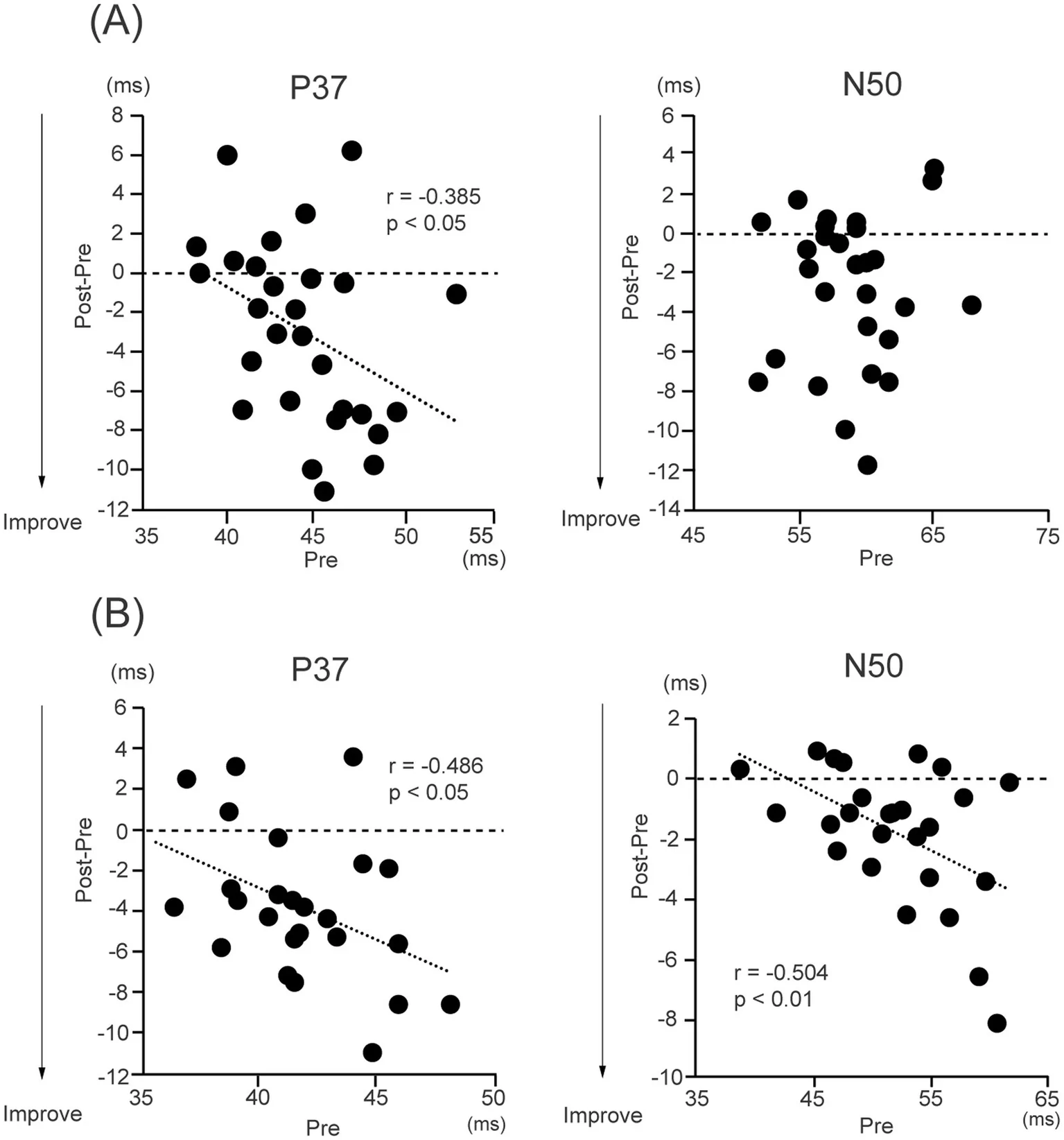
Correlation of latency change between pre- and post-intervention during **(A)** tibial nerve stimulation; **(B)** peroneal nerve stimulation in PES. PES, plantar electrical stimulation group.

### 3.7 Stimulus intensity during SEP recording

ANOVAs for the intensity of electric stimulation showed a significant main effect of Time during peroneal nerve stimulation (*F* (1, 52) = 15.238, *p* < 0.001, *η*^2^ = 0.227), indicating that the stimulus intensity was higher in Pre than Post (Table 1). No other significant difference was observed. In addition, no significant correlations were identified between baseline stimulus intensity and baseline SEP latencies for any of the SEP components that showed intervention-related effects.

To further examine whether changes in stimulation intensity were associated with changes in SEP latency, correlation analyses were conducted between changes in stimulation intensity (Post-Pre) and changes in P37 and N50 latencies (Post-Pre) separately for the PES and CON groups. For tibial nerve stimulation, no significant correlations were observed in either the PES group (P37: *r* = −0.122, *p* = 0.546) or the CON group (P37: *r* = 0.250, *p* = 0.209). Similarly, for peroneal nerve stimulation, no significant correlations were observed in either the PES group (P37: *r* = −0.064, *p* = 0.745; N50: *r* = −0.016, *p* = 0.935) or the CON group (P37: *r* = −0.049, *p* = 0.806; N50: *r* = 0.348, *p* = 0.069).

### 3.8 Associations between intervention dosage and changes in SEP latency

To examine whether intervention dosage was associated with changes in SEP latency, correlation analyses were performed separately for tibial and peroneal nerve stimulation using participants included in each SEP analysis. In the tibial nerve analysis, neither the total number of intervention sessions nor the average stimulation intensity was significantly correlated with changes in P37 latency (*p* > 0.05). Similarly, in the peroneal nerve analysis, no significant correlations were observed between either intervention dosage measure and changes in P37 or N50 latency (all *p* > 0.05).

## 4 Discussion

The present study examined the effects of plantar stimulation on somatosensory processing, as assessed by SEP latencies and amplitudes elicited by tibial, peroneal, and median nerve stimulation. The principal finding was that 8 weeks of home-based plantar electrical stimulation was associated with shortened latencies of early SEP components elicited by both tibial and peroneal nerve stimulation, whereas no comparable changes were observed in the control group or upper-limb SEPs. Significant negative correlations were also noted between baseline latencies and intervention-related changes, although these correlations should be interpreted cautiously because of mathematical coupling between baseline values and change scores and the possibility of regression to the mean. In contrast, no significant intervention-related changes were identified in SEP amplitudes during lower-limb stimulation. Taken together, these findings provide neurophysiological evidence that repeated plantar electrical stimulation may be associated with changes in the temporal characteristics of lower-limb somatosensory processing in older adults.

The observed shortening of the P37 latency during tibial nerve stimulation and P37 and N50 latencies during peroneal nerve stimulation is consistent with changes in the temporal characteristics of neural responses to lower-limb somatosensory input. The persistence of the group effect for P37 latency after adjustment for baseline differences suggests that baseline variability alone is unlikely to fully account for the observed latency shortening. In contrast, although tibial N50 latency was also shortened in the repeated-measures ANOVA, this effect was no longer significant after adjustment, indicating that baseline differences may have contributed to the observed change. The P37 component represents one of the earliest cortical responses elicited by lower-limb afferent input and is considered to primarily reflect activation of the primary somatosensory cortex. In contrast, the subsequent N50 component is considered to involve a broader sensorimotor integration network extending beyond the primary somatosensory cortex (Valeriani et al., 2001). Therefore, the consistent shortening of the P37 latency observed following both tibial and peroneal nerve stimulation may reflect changes in the early stages of lower-limb somatosensory processing following repeated plantar electrical stimulation. However, it should be noted that the electrical stimulation used to elicit SEPs was adjusted to the motor threshold and produced a visible motor response. Therefore, the recorded responses may reflect not only somatosensory afferent processing but also motor-related or sensorimotor integration processes. The present findings should therefore not be interpreted as reflecting changes in isolated somatosensory processing alone.

Aging is associated with slower peripheral nerve conduction and prolonged SEP latencies (Jacobs and Love, 1985; Kakigi, 1987). Because lower-limb somatosensory input plays an essential role in postural control and locomotion, the observed changes in somatosensory processing may have functional relevance for sensorimotor control in older adults; however, their functional significance remains unclear because functional outcomes were not directly assessed in the present study. Previous intervention studies reported that somatosensory exercise can improve balance and physical performance in older adults (Sedighi Darijani et al., 2024), suggesting the potential functional relevance of somatosensory interventions in this population. However, whether the neurophysiological changes observed in the present study are associated with such functional improvements remains to be determined. The present findings therefore may reflect changes in the temporal characteristics of neural processing within lower-limb somatosensory pathways associated with repeated plantar electrical stimulation.

Furthermore, significant negative correlations between baseline SEP latency (Pre) and intervention-related changes in latency (Post-Pre) were observed for the P37 component during tibial nerve stimulation, as well as for the P37 and N50 components during peroneal nerve stimulation. Thus, participants with longer baseline latencies tended to exhibit greater reductions in latency following the intervention (Figure 6). These findings indicate an association between baseline SEP latency and the magnitude of subsequent latency change. The possibility that the effects of somatosensory training depend on the initial functional state was suggested in a systematic review by Sasaki et al. (2025), who collated findings from multiple previous studies, and the present results are broadly consistent with these observations. However, because change scores mathematically include baseline values, the observed negative correlations may partly reflect mathematical coupling rather than a true physiological relationship. Therefore, these findings should be interpreted with caution and require confirmation in future studies using analytical approaches that are less susceptible to baseline-dependent bias.

In addition, a significant reduction in stimulus intensity was observed during peroneal nerve stimulation in both the PES and CON groups, whereas no significant change was found during tibial nerve stimulation. Although previous studies reported nerve-specific differences in SEP responses to stimulation parameters (Onishi et al., 1991), the mechanism underlying the reduction in stimulation intensity observed specifically during peroneal nerve stimulation remains unclear. This finding suggests that neural responses associated with peroneal nerve stimulation may be more susceptible to repeated sensory input. However, given the absence of a Time × Group interaction, the present findings do not allow us to conclude that these peripheral changes were specifically induced by the intervention. Furthermore, no significant correlations were observed between the baseline stimulation intensity and baseline SEP latencies for the components that showed intervention-related effects. This finding suggests that the baseline differences in SEP latency were unlikely to be explained by variations in stimulation intensity. In addition, changes in stimulation intensity were not significantly correlated with changes in P37 or N50 latency during either tibial or peroneal nerve stimulation in either group. Thus, although the potential influence of stimulation intensity cannot be completely excluded, these findings do not support a clear association between changes in stimulation intensity and the observed SEP latency changes.

Individual variability in adherence to the home-based intervention should also be considered when interpreting the present findings. Although 73.0% of participants in the PES group met the minimum protocol criterion of completing at least two stimulation sessions per week during each of the eight intervention weeks, the total number of sessions varied considerably among participants. However, neither the total number of intervention sessions nor the average stimulation intensity was significantly correlated with changes in P37 or N50 latency following tibial or peroneal nerve stimulation. Thus, the present findings do not provide evidence of a clear dose–response relationship between intervention dosage and SEP latency changes. Nevertheless, the potential influence of individual variability in adherence cannot be completely excluded.

Changes were also observed in SEP components elicited by median nerve stimulation. The N60 latency at C3’ was prolonged following the intervention in the PES group, whereas the N27 amplitude was reduced over time in both groups (Table 5). Although the N27 component reflects early cortical processing within the primary somatosensory cortex, and the N60 component has been associated with broader sensorimotor integration networks (Barba et al., 2001, 2008), the physiological significance of these findings remains unclear because they were inconsistent with the latency shortening observed in the lower-limb SEP. Therefore, the absence of comparable latency shortening in median nerve SEPs is consistent with the possibility that the observed changes were more prominent in lower-limb somatosensory processing than in upper-limb processing. However, this finding alone does not establish lower-limb-specific neural adaptations.

This study had several limitations. First, although significant reductions in SEP latencies were observed following the intervention, the underlying neural mechanisms remain unclear. The present study relied on scalp-recorded SEPs, which provide high temporal resolution but limited spatial specificity. Therefore, it is difficult to determine the precise cortical and subcortical structures contributing to the observed latency changes. Future studies combining SEP with neuroimaging techniques, such as fMRI, may help clarify the neural sources and networks involved. Second, although significant intervention effects were observed, the sample size was relatively modest and baseline differences contributed to some of the observed changes, particularly for the tibial N50 component. Although ANCOVA was used to adjust for baseline differences, the possibility of regression to the mean cannot be completely excluded. In addition, the observed negative correlations between baseline latency and intervention-related changes should be interpreted cautiously because change scores mathematically include baseline values. Larger studies are needed to confirm these findings, improve their generalizability, and better characterize variability in neurophysiological responsiveness to plantar electrical stimulation. Third, the stimulation was performed by participants at home, which limited the degree of experimental control. Although standardized instructions were provided regarding electrode placement and stimulation procedures, minor variations in stimulation location, intensity, and timing may have occurred across individuals and sessions because participants selected a comfortable stimulation intensity and performed the intervention at their preferred time of day. Future studies conducted under more tightly controlled conditions are needed to determine the optimal stimulation parameters and verify the reproducibility of the present findings. Fourth, the present study included a no-intervention control group but did not include a sham stimulation group. Therefore, the specific effects of plantar electrical stimulation cannot be completely distinguished from nonspecific effects associated with participation in the intervention. Future studies incorporating a sham stimulation condition are needed to clarify the specific effects of plantar electrical stimulation. In addition, participants were allocated to the PES or CON group based on their willingness to undertake the eight-week intervention rather than through random assignment. Therefore, selection bias cannot be excluded, and unmeasured differences in motivation or other characteristics between the groups may have influenced the results. Future studies using randomized allocation are needed to confirm the present findings. Finally, the present study did not include direct behavioral or functional measures, such as gait or balance assessments. Future studies should determine whether the observed neurophysiological changes are associated with improvements in mobility, balance, and other functional outcomes in older adults.

## 5 Conclusion

The present study showed that 8 weeks of home-based plantar electrical stimulation was associated with shortened latencies of early SEP components elicited by tibial and peroneal nerve stimulation in older adults. In contrast, no comparable latency shortening was observed in median nerve SEPs, suggesting that the observed neurophysiological changes were more prominent in lower-limb than in upper-limb somatosensory processing. These findings suggest that repeated plantar electrical stimulation may be associated with changes in the temporal characteristics of lower-limb somatosensory processing. Further studies are warranted to clarify the underlying mechanisms and determine whether these neurophysiological changes are associated with improvements in balance, mobility, and other functional outcomes in older adults.

## Data Availability

The raw data supporting the conclusions of this article will be made available by the authors, without undue reservation.
